# Maintenance of self-incompatibility in peripheral populations of a circumboreal woodland subshrub

**DOI:** 10.1093/aobpla/plu063

**Published:** 2014-10-20

**Authors:** Ai-Qin Zhang, Ying-Ze Xiong, Shuang-Quan Huang

**Affiliations:** 1 College of Life Science and Technology, Xinjiang University, Urumqi 830046, China; 2 School of Life Sciences, Central China Normal University, Wuhan 430079, China

**Keywords:** Baker's law, clonality, geitonogamy, generalist pollinator, marginal distribution, self-compatible, self-incompatible, stigmatic pollen load.

## Abstract

Compared with self-incompatible (SI) species, species that shift to self-compatibility (SC) are more likely to colonize a new habitat. Self-incompatibility and fruit-set failure have been widely reported in European populations of *Linnaea borealis* (twinflower), whereas at the eastern margin of its North American distribution it showed potential SC. We investigated the breeding system of *L. borealis* in northwestern China, the eastern margin of the species' distribution in Eurasia. Pollinators, breeding system and pollen limitation were examined in a nature reserve with thousands of *L. borealis* individuals. To investigate whether fruit set was limited by mating opportunity, we compared fruit set in high-, medium- and low-density patches of *L. borealis*. To examine whether clonal reproduction resulted in higher fruit-set failure, we compared fruit set among different sizes of clonal ramets. Flies contributed most pollinator visits in the studied population. It was strictly SI and natural fruit set depended on insect visits. Patch density comparisons showed that *L. borealis* was not pollen limited in low-density patches that had significantly fewer flowers. However, it produced significantly fewer fruits per flower when clonal ramet size increased, suggesting that the high failure of fruit set in larger clones with more flowers may be caused by geitonogamy. Generalist pollinators and clonal reproduction may help *L. borealis* to colonize in marginal areas without the transition of the breeding system from SI to SC, but experiencing fruit-set failure resulting from geitonogamy within clones.

## Abstract (precis)

Peripheral populations of plant species are more likely to experience a shift of breeding system from self-incompatible (SI) to self-compatible (SC). *Linnaea borealis* named after Carl Linnaeus, commonly known as twinflower, is an undershrub of woods with a circumpolar distribution in boreal forests. We observed that it was strictly SI in northwestern China, the eastern margin of the species' distribution in Eurasia. Generalist pollinators and clonal reproduction may help *L. borealis* to colonize in marginal areas without the shift from SI to SC, but experiencing fruit-set failure resulting from geitonogamy (intra-plant pollination) within clones.

## Introduction

Self-incompatibility is well established as a physiological mechanism to avoid self-fertilization and prevent inbreeding depression in plants (de Nettancourt 1977; Matton *et al.* 1999; Castric and Vekemans 2004). Baker (1955) noted that self-compatible (SC) species were more likely to colonize new habitats after long-distance dispersal than self-incompatible (SI) ones. Theoretical consideration of the evolutionary transition from SI to SC indicates that it can be caused by a single factor, selection for reproductive assurance (Lloyd 1992; Lloyd and Schoen 1992; Herlihy and Eckert 2002; Barrett 2008; Busch and Delph 2012). For example, in Himalayan mayapple (*Podophyllum hexandrum*), the shift from SI to SC could be adaptive when pollinators are scarce as a means to achieve reproductive success through automatic self-pollination (Xiong *et al.* 2013). A large number of studies have observed an association between self-fertilization and colonizing ability in various contexts (Cheptou 2012), in support of Baker's law. However, an increasing body of literature reveals exceptions to Baker's law (Carr *et al.* 1986; Sun and Ritland 1998; Brennan *et al.* 2005, 2006; Miller *et al.* 2008). For example, maintenance of SI was observed in two African species of *Lycium* (Solanaceae), a genus that originated in South America and dispersed to the Old World on a single occasion (Miller *et al.* 2008). Study of the exceptions to Baker's law could provide more insights into the transition of breeding systems between SI and SC.

*Linnaea borealis* (Caprifoliaceae or Linnaeaceae) is an undershrub of woods with a circumpolar distribution in boreal forests across North America, Europe and Asia (Wilcock 2002). The species, commonly known as twinflower, is named after Carl Linnaeus. It is capable of vegetative reproduction by stolons as well as sexual reproduction. Previous studies have shown widespread sexual reproductive failure in populations from central Alberta in Canada (Ross and La Roi 1990) through east Aberdeenshire in Britain (Wilcock and Jennings 1999) and Cairngorms National Park (Scobie and Wilcock 2009) to central Sweden, Scandinavia (Eriksson 1992). Self-pollination did not produce seeds in *L. borealis*, suggesting that it was completely SI (Wilcock and Jennings 1999). However, an investigation of the breeding systems of boreal forest herbs in New Brunswick, NJ, USA, at the eastern edge of the species distribution in North America, showed that fruit set and seed set were not significantly different between self- and cross-pollination, suggesting SC in *L. borealis* (Barrett and Helenurm 1987). A recent comprehensive study of reproductive ecology in *L. borealis* in Scotland showed self-pollination resulting in very low fruit set (Scobie and Wilcock 2009). This geographical variation in the capability of SI in *L. borealis* suggests that peripheral populations may evolve from SI to SC if reproduction is pollen limited.

To better understand diversification of the breeding system across geographical regions, we investigated insect pollination and the breeding system of *L. borealis* in northwest China, at the eastern edge of its Eurasian distribution. We address the following questions: (i) is *L. borealis* SI or SC in this peripheral population? (ii) Is it pollen limited? Is there severe fruit-set failure? Particularly, we compared fruit set at different patch densities and genet sizes to examine whether mating opportunity limitation and vegetative reproduction affect reproductive success. This investigation permits us to examine variation in the breeding system across the distribution of this species and to test Baker's law at the margin of its distributional range.

## Methods

### The study species

*Linnaea borealis* L. (Caprifoliaceae, or Linnaeaceae) is a rare, clonal dwarf species belonging to a monotypic genus, generally boreal to the subarctic woodland subshrub. It occupies a small area in East Asia including Xinjiang, inner Mongolia and northeastern China. Flowering occurs from early June to late July. Each inflorescence consists of a pair (occasionally three or four) of small white/pink campanulate, sweetly scented flowers. Each ﬂower has four didynamous stamens, two of which are higher than the others, and one pistil with a three-celled ovary containing ∼10 ovules (Barrett and Helenurm 1987). The flowers last for 4–5 days and stamens dehisce soon after the flower opens. The ovary ripens in late July producing a single-seeded fruit (Scobie and Wilcock 2009). The field population was studied in Xinjiang Altai Kanas Nature Reserve (48°42′51″N, 87°01′35″E, 1383 m above sea level), Xinjiang Province, northwest China.

Flower-visiting insects were observed in 6 fixed patches in 2012 and 13 patches in 2013 with ∼100 flowers per patch for a period of 30 min at a time. A total of 28 h on sunny days during July 2012 and 2013 were spent watching flower-visiting insects.

### Pollination treatment

To examine the breeding system in *L. borealis*, we randomly selected 25 individuals (genets) and bagged flower buds with fine nylon mesh nets to exclude flower visitors before treatments. Four treatments were conducted on each individual as follows: (i) flowers were bagged during anthesis to exclude pollinators, and to examine possible automatic self-pollination (*n* = 52); (ii) flowers were hand-pollinated with self-pollen to examine SC (*n* = 66); (iii) flowers were hand-pollinated with pollen from other plants at least 10 m away (*n* = 50); and (iv) stamens were removed and the flowers were bagged during anthesis to examine the possibility of apomixis (*n* = 36). When fruits ripened, the fruit set of additional 477 natural flowers was surveyed as a control. We compared fruit set per flower among different treatments using the non-parametric Kruskal–Wallis test and used the Mann–Whitney test for comparisons between every two treatments.

### Natural pollination level

To investigate the natural levels of pollination, we recorded the natural fruit set and stigmatic pollen load in 2013. The two longer stamens in *L. borealis* usually dehisced first, before the two shorter ones. Thirty flowers at the two- and four-stamen dehiscence stage were randomly collected and stigmatic pollen grains from these flowers were counted under a microscope. The percentage of these 30 stigmas with pollen loads at late anthesis (all anthers dehisced) was also recorded and compared with the natural fruit set using one-sample *t*-tests to examine the possibility of mating opportunity limitation. To investigate automatic self-pollen deposition in *L. borealis*, we randomly labelled 30 flower buds and bagged them to exclude pollinator visitation during anthesis until the flowers wilted. Samples of 376, 612 and 660 flowers were checked for natural fruit set in another three patches in the study site.

### Effects of patch density and ramet size on fruit set

To examine whether the fruit set in *L. borealis* was limited by mating opportunity, we randomly selected 30 high-, medium- and low-density patches (0.5 m × 0.5 m) according to the number of ramets (∼45, 30 and 15 ramets, respectively) in each patch and the number of sexual (flowering) branches and the fruit set in each patch. The number of sexual branches and the fruit set were compared using one-way ANOVA and least-significant difference (LSD) post-hoc tests.

To investigate whether large clones resulted in increased self-pollination within individuals (i.e. geitonogamy) and higher fruit failure, we randomly labelled 30 large, medium-sized and small clonal ramets (ramet length: <50, 50–80, >80 cm). When the fruit ripened, mean flower and fruit number per ramet were recorded. Flower and fruit number and fruit set were compared among three ramet sizes using one-way ANOVA and LSD post-hoc tests.

### Data analysis

All data analysis was conducted in SPSS, version 17.0. All means are presented with standard errors (±SE).

## Results

### Pollinator observations

Our 28 h of observation recorded diverse insects including solitary bees (Halictidae), hoverflies (Syrphidae) and flies (Anthomyiidae, Muscidae) visiting flowers of *L. borealis*. Flies contributed nearly 70 % of the total visits, indicating that flies were the most frequent visitors for this species. Visitation frequencies of the three types of insects were 0.083 ± 0.042, 0.058 ± 0.060, 0.158 ± 0.042 and 0.018 ± 0.001, 0.015 ± 0.009, 0.053 ± 0.008 visits per flower per hour in 2012 and 2013, respectively.

### Breeding system

**Figure 1.**
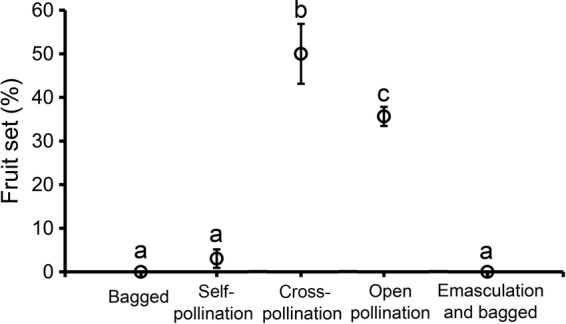
A comparison of fruit set under five treatments (flowers bagged, hand self-pollination, hand cross-pollination, open pollination and flowers bagged after emasculation). Kruskal–Wallis test, *χ*^2^ = 79.14, df = 4, *P* < 0.001, significant differences between means by the Mann–Whitney test are indicated by different letters.

Natural fruit set per ramet in *L. borealis* was 35.64 ± 2.20 % in the study site. Fruit set of bagged flowers (autogamy) and bagged flowers after emasculation (apomixis) were negligible (Fig. 1), indicating the absence of automatic self-pollination and apomixis. Self-pollinated flowers set very few fruits; their fruit set was 3.03 ± 2.13 %, significantly lower than the fruit set under cross-pollination (50.00 ± 6.87 %) (*Z* = −5.88, *P* < 0.001, Fig. 1). The results indicated that *L. borealis* was SI in the study area. Fruit set after cross-pollination was significantly higher than that after natural pollination (*Z* = −2.069, *P* = 0.039, Fig. 1), indicating that sexual reproduction in *L. borealis* was pollen limited.

### Natural pollination level

All three patches in our study site showed considerable fruit set ranging from 22.71 ± 1.69 to 43.09 ± 2.56 %. Pollen grain counts on the stigma increased after the stamens dehisced (two stamens dehisced: 13.2 ± 2.87; four stamens dehisced: 19.2 ± 3.0), while no pollen grains were observed on the stigma when flowers were bagged throughout the flowers' lifetime, suggesting that there was no automatic self-pollination while the flower was open. This result is consistent with the failure of fruit set of bagged flowers. Stigmas (84.2 %) had pollen on them after all stamens had dehisced, which was significantly higher than the final fruit set (*t* = 15.85, df = 476, *P* < 0.001), indicating that *L. borealis* received numerous pollinator visits during anthesis but that fruit production was significantly limited by the availability of compatible pollen.

### Effects of ramet size and patch density on fruit set

**Figure 2.**
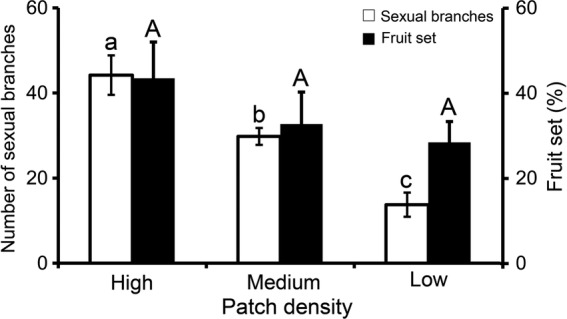
Number of sexual branches (open bars) and fruit set (closed bars) comparisons among three different patch densities (high, medium and low). One-way ANOVA, *F*_2, 87_ = 20.71, *P* < 0.001 and *F*_2, 87_ = 1.18, *P* = 0.312 for branch number and fruit set, respectively. Significant differences between means are indicated by different letters.

**Figure 3.**
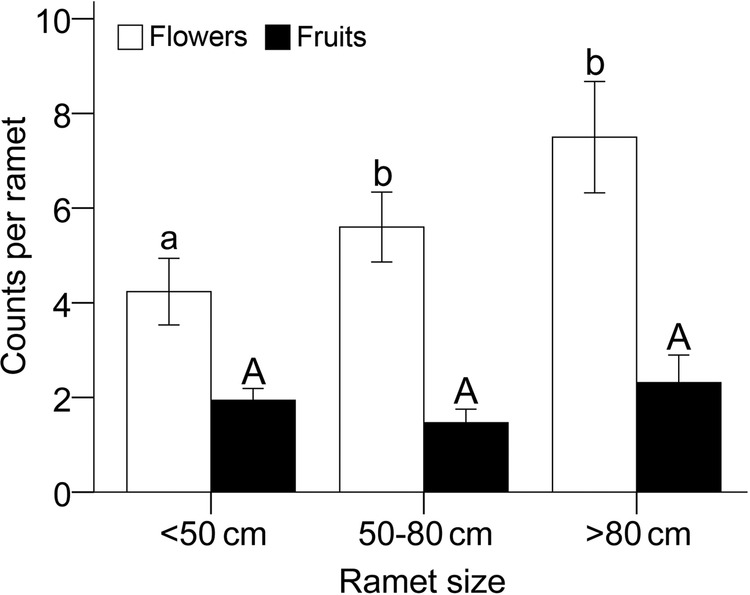
Flower (open bars) and fruit production (closed bars) comparisons among three different ramet sizes (<50, 50–80 and >80 cm). One-way ANOVA, *F*_2, 60_ = 3.13, *P* = 0.05 and *F*_2, 60_ = 1.34, *P* = 0.271 for flower and fruit production, respectively. Significant differences between means using LSD tests are indicated by different letters.

Patch density and ramet size showed different effects on fruit set. Although the number of sexual branches increased with patch density (*F*_2, 87_ = 20.71, *P* < 0.001, Fig. 2), fruit set did not differ significantly with patch density (*F*_2, 87_ = 1.18, *P* = 0.312, Fig. 2). Flower number increased significantly with ramet size (*F*_2, 60_ = 3.13, *P* = 0.05), but fruit number did not (*F*_2, 60_ = 1.34, *P* = 0.271). As a result, the smallest ramets (<50 cm) had significantly higher ratios of fruit set per ramet than larger ramets (50–80 and >80 cm, *Z* = 0.25 and 0.30, *P* = 0.012 and 0.007, respectively, Fig. 3).

## Discussion

Our pollination treatments confirmed that *L. borealis* is SI and that fruit set depends on insect pollination in this field population at the margin of its distribution. To a certain degree, the fruit-set failure could be attributed to geitonogamy among clonal ramets. Flies were the primary visitors to *L. borealis* as was the case in the north-east of Scotland (Scobie and Wilcock 2009). Stigmatic pollen deposition and flower bagging experiments showed no automatic self-pollination during anthesis. The hand self-pollination experiment indicated that this population of *L. borealis* rarely sets fruits, suggesting that it was SI. Lower patch density did not significantly lower fruit set, indicating that compatible mates were not limiting among genets in the study population. These results are consistent with a recent study in Scotland (Scobie and Wilcock 2009). However fruit set per ramet significantly decreased when the ramet size was larger, suggesting that geitonogamous pollination within ramets significantly affected sexual reproduction.

Most previous studies have shown that *L. borealis* was SI. An earlier work by Wilcock and Jennings (1999) found it to be completely SI, with no fruit set under self-pollination. In a later work, Scobie and Wilcock (2009) observed that *L. borealis* could still set a few fruits when receiving only self-pollen. They attributed the former result to small samples (Scobie and Wilcock 2009). Self-compatible species are more likely to colonize a new habitat than SI ones (Lloyd 1992). *Linnaea borealis* has a circumpolar distribution in boreal forests across North America, Europe and Asia (Wilcock 2002). Our study site is located at the margin of its distribution in East Asia. The result of our breeding system experiment indicated that *L. borealis* at this marginal site was still highly SI, although the extremely low fruit set after self-pollination indicated leaky self-incompatibility. Leaky self-incompatibility has been observed in several other species (e.g. Les *et al.* 1991; Reinartz and Les 1994; Luijten *et al.* 1996) and may represent a partial breakdown of self-incompatibility (Reinartz and Les 1994). Interestingly in another work in North America, Barrett and Helenurm (1987) observed that both fruit set and seed set showed no significant difference between self- and cross-pollination, indicating the capacity for SC in *L. borealis*.

On the other hand, the maintenance of SI in *L. borealis* could be attributed to clonal reproduction. In SI species, clonal reproduction allows the foundation and maintenance of a population in an unpredictable pollination environment (under pollen limitation). Vallejo-Marín and O'Brien (2007) and Vallejo-Marín (2007) suggested that avoidance of geitonogamy played a significant role in the maintenance of SI in clonal plants. An increasing body of literature shows widespread sexual reproductive failure in *L. borealis*, across its distributional range (Jackson 1939; Polunin 1959; Clapham *et al.* 1962; Ross and La Roi 1990; Eriksson 1992; Wilcock and Jennings 1999; Scobie and Wilcock 2009). Our hand cross-pollination experiment also clarified the existence of pollen limitation in the species (Fig. 1). Although our study showed little limitation of mating opportunity between genets (Fig. 2), avoidance of geitonogamous pollination within genets (Fig. 3) could be a significant factor maintaining SI in *L. borealis*.

Fecundity has been shown to be reduced in small patches of the clonal *Calystegia collina* (E. Greene) Brummitt, which is primarily SI and bee pollinated. Reproductive failure was attributed to the greater distances to the nearest compatible individual for plants in small patches compared with those in larger ones (Wolf and Harrison 2001). In our study low-density patches produced significantly fewer flowers than high-density ones which may limit the availability of compatible pollen, but fruit set did not decrease in low-density patches (Fig. 2). This could be attributed to pollinator behaviour. A wide diversity of insect visitors to *L. borealis* was observed in Scotland (Wilcock and Jennings 1999) and Canada (Barrett and Helenurm 1987). The species is generalist pollinated and the principal visitors were small insects including hoverflies and other flies. The extent of geitonogamy may be affected by pollinator behaviour, for example, smaller insects having shorter flight distances than larger ones (Westerbergh and Saura 1994; Scobie and Wilcock 2009). In our study site, *L. borealis* was probably primarily pollinated by flies that have limited pollen-dispersal capabilities, aggravating the difficulty of pollen exchange between compatible mates. Scobie and Wilcock (2009) observed higher pollen loads as patch density increased, but did not observe a significant relationship between fruit set and patch density. Although plants in large patches may have more compatible flowers to mate with and be more attractive to pollinators, the low efficiency of pollen exchange between mates may result in incompatible pollen discounting.

As SI plants require cross-pollination with a compatible mate, scarcity of available pollen could be severe in clonal SI plants. As clonal plants spread, individuals become increasingly surrounded by flowers of the same genet, and self-pollination between them (geitonogamy) becomes more likely (Handel 1985; Charpentier 2002; Araki *et al.* 2007). In isolated monoclonal patches, geitonogamy is inevitable and will result in total reproductive failure (Wilcock and Jennings 1999). A negative correlation between ramet size and seed set was found in mixed populations of a self-incompatible clonal plant (Laverty and Plowright 1988), a situation that we suspect also occurred in *L. borealis* at our study site (Fig. 3). In the larger clones, individual flowers are more likely to be surrounded by inflorescences of the same plant, resulting in more geitonogamous pollination, after which fruit set will be unlikely in SI species.

Our study provides additional evidence contributing to our understanding of why there is no shift of the breeding system from SI to SC in peripheral populations of *L. borealis*. In this shift, reproductive assurance may play a major role in driving SI to SC (Busch and Delph 2012). The generalist pollinators and the capacity for clonal reproduction permit a species to invade new habitats in an environment where reproduction by seeds is pollen limited (Vallejo-Marín 2007).

## Sources of Funding

This work was supported by the National Science Foundation of China (NSFC no. U1203102; 31030016).

## Contributions by the Authors

A.-Q.Z. conducted 2 years of fieldwork and Y.-Z.X. and S.-Q.H. joined in 1 year. Y.-Z.X. and S.-Q.H. wrote the manuscript. All authors contributed to experimental design and data analysis and commented on the manuscript.

## Conflicts of Interest Statement

None declared.
